# Hyperfocus in ADHD: A Misunderstood Cognitive Phenomenon

**DOI:** 10.1192/j.eurpsy.2025.662

**Published:** 2025-08-26

**Authors:** B. A. Oroian, P. Nechita, A. Szalontay

**Affiliations:** 1 “Grigore T. Popa” University of Medicine and Pharmacy Iasi; 2 ” Socola” Institute of Psychiatry Iasi, Iasi, Romania

## Abstract

**Introduction:**

Attention-Deficit/Hyperactivity Disorder (ADHD) is characterized by deficits in attention, impulsivity, and hyperactivity, but some cognitive phenomena within ADHD remain under-researched. One such phenomenon is hyperfocus, referring to prolonged, intense concentration on tasks of high interest. While inattentiveness is a hallmark of ADHD, hyperfocus presents a paradox where individuals become deeply absorbed in activities, often neglecting other necessary responsibilities. This focus can lead to productive work but also negative outcomes like missed deadlines or personal neglect.

**Objectives:**

This study investigates the prevalence of hyperfocus in adults with ADHD, explores its relationship to core symptoms, and examines its impact on functional outcomes, including academic performance, employment, and relationships. It also seeks to understand individuals’ perceptions of hyperfocus’s benefits and drawbacks.

**Methods:**

A mixed-methods approach was used to capture both quantitative and qualitative data. A total of 50 adults diagnosed with ADHD, aged between 18 and 45, were recruited. Participants completed the ADHD-Focused Attention Questionnaire (AFAQ), designed to measure the frequency, intensity, and duration of hyperfocus episodes. Additionally, the participants were asked to complete a structured life events questionnaire, assessing the impact of hyperfocus on various functional domains, including academic performance, employment, and personal relationships.

**Results:**

The study found that 68% of participants reported frequent hyperfocus, with episodes lasting from several hours to days. The most common triggers were work-related tasks (35%), creative activities (25%) and gaming (20%),. While hyperfocus during gaming and creative tasks brought personal satisfaction, it often resulted in neglected responsibilities (40%). Hyperfocus at work increased productivity for 30% of participants, particularly in flexible or creative roles. There was also a strong correlation between hyperfocus and productivity, especially in flexible environments. However, hyperfocus also correlated with missed deadlines and neglected self-care, especially in less structured routines. Interviewees highlighted hyperfocus as a double-edged sword: it provided intense concentration but left many feeling “trapped,” struggling to shift attention. Regarding relationships, 55% of participants said hyperfocus negatively impacted their social lives, with partners feeling neglected. However, 15% found that hyperfocus occasionally enhanced shared activities.

**Conclusions:**

Hyperfocus in ADHD boosts productivity but can disrupt routines and relationships. Its unpredictability complicates balancing responsibilities. Recognizing hyperfocus as a core feature in ADHD could lead to better management strategies, such as time management training, external reminders, and structured breaks.

**Disclosure of Interest:**

None Declared

